# Repositioning chlorpromazine for treating chemoresistant glioma through the inhibition of cytochrome c oxidase bearing the COX4-1 regulatory subunit

**DOI:** 10.18632/oncotarget.17247

**Published:** 2017-04-19

**Authors:** Claudia R. Oliva, Wei Zhang, Cathy Langford, Mark J. Suto, Corinne E. Griguer

**Affiliations:** ^1^ Department of Neurosurgery, University of Alabama at Birmingham, Birmingham, 35294 Alabama, USA; ^2^ Southern Research, Birmingham, 35294 Alabama, USA; ^3^ Center for Free Radical Biology, University of Alabama at Birmingham, Birmingham, 35294 Alabama, USA

**Keywords:** cytochrome c oxidase, chlorpromazine, glioblastoma, inhibitor, stem cells

## Abstract

Patients with glioblastoma have one of the lowest overall survival rates among patients with cancer. Standard of care for patients with glioblastoma includes temozolomide and radiation therapy, yet 30% of patients do not respond to these treatments and nearly all glioblastoma tumors become resistant. Chlorpromazine is a United States Food and Drug Administration-approved phenothiazine widely used as a psychotropic in clinical practice. Recently, experimental evidence revealed the anti-proliferative activity of chlorpromazine against colon and brain tumors. Here, we used chemoresistant patient-derived glioma stem cells and chemoresistant human glioma cell lines to investigate the effects of chlorpromazine against chemoresistant glioma. Chlorpromazine selectively and significantly inhibited proliferation in chemoresistant glioma cells and glioma stem cells. Mechanistically, chlorpromazine inhibited cytochrome c oxidase (CcO, complex IV) activity from chemoresistant but not chemosensitive cells, without affecting other mitochondrial complexes. Notably, our previous studies revealed that the switch to chemoresistance in glioma cells is accompanied by a switch from the expression of CcO subunit 4 isoform 2 (COX4-2) to COX4-1. In this study, chlorpromazine induced cell cycle arrest selectively in glioma cells expressing COX4-1, and computer-simulated docking studies indicated that chlorpromazine binds more tightly to CcO expressing COX4-1 than to CcO expressing COX4-2. In orthotopic mouse brain tumor models, chlorpromazine treatment significantly increased the median overall survival of mice harboring chemoresistant tumors. These data indicate that chlorpromazine selectively inhibits the growth and proliferation of chemoresistant glioma cells expressing COX4-1. The feasibility of repositioning chlorpromazine for selectively treating chemoresistant glioma tumors should be further explored.

## INTRODUCTION

Temozolomide (TMZ), an alkylating agent that has shown significant initial benefit in the treatment of high-grade gliomas, especially when combined with radiotherapy, is commonly used in the adjunctive treatment of gliomas. However, TMZ chemotherapy eventually becomes impaired by the development of chemoresistance. Indeed, this phenomenon presents the most challenging barrier in the successful treatment of cancer and is the principal reason for chemotherapy failure and one of the main reasons underlying the failure to demonstrate a sustainable beneficial clinical outcome for patients with glioblastoma (GBM) [1, 2].

Differentiated bulk tumor cells commonly use less efficient glycolysis for the production of ATP (Warburg effect) [3]. However, tumors also contain cancer stem cells (CSCs), a subset of cancer cells that have the ability to repopulate the entire tumor and thus lead to recurrence. Distinct metabolic phenotypes have been described for CSCs, depending on the cancer type [4]. The concept of glycolysis-driven CSCs has been demonstrated in breast cancer [5], nasopharyngeal carcinoma [6], and hepatocellular carcinoma [7]. Conversely, CSCs driven by mitochondrial oxidative phosphorylation (OxPhos) have been identified in lung cancer [8, 9], glioma [10–12], pancreatic cancer [13], and leukemia stem cells [14]. Regardless of the primary metabolic phenotype, however, mitochondrial function appears to be critical for CSC functionality, and elimination of highly chemoresistant CSCs via inhibition of mitochondrial function may prevent relapse from disease and thus improve patients’ long-term outcome [4].

Cytochrome c oxidase (CcO) is the terminal enzyme of the mitochondrial respiratory chain (electron transport chain, ETC) that catalyzes the transfer of electrons from cytochrome c (cyt c) to oxygen (O_2_). Mammalian CcO is a complex enzyme that comprises three mitochondrial DNA-encoded subunits that perform the catalytic function and 10 nuclear-encoded subunits that regulate the catalytic activity [15, 16]. CcO activity governs the electron flux capacity of the ETC, thus controlling the efficiency of mitochondrial coupling and thereby the production of reactive oxygen species (ROS) [12, 17–19].

Several studies have focused on the biological functions of CcO subunits in the development of tumors. Chen et al. reported that knockdown of CcO subunit 5a (COX5a) expression substantially suppresses the migration and invasion of non-small cell lung cancer cells through inhibition of metalloproteinases MMP-2 and MMP-9 [20]. Moreover, elevated COX5a expression is associated with higher N stage (a parameter that indicates cancer spread to the nearby lymph nodes) and poorer prognosis of patients with lung adenocarcinoma. Additionally, COX5b is involved in the metastatic potential of colorectal carcinoma cells [21]. Notably, CcO subunit isoform expression can vary by tissue and developmental stage [22] and may also regulate CcO activity. In GBM, increased CcO activity and increased expression of CcO subunit 4 isoform 1 (COX4-1) have been associated with acquisition of TMZ chemoresistance [12] as well as shorter progression-free and overall survival of patients [23].

COX4 performs an essential regulatory role in CcO by binding ATP (allosteric inhibitor) and ADP (allosteric activator), thereby adjusting energy production to energy demand [24–27]. These alterations are likely to facilitate adaptive chemoresistance through the suppression of apoptotic signaling [28]. Indeed, inhibition of CcO activity or decreased expression of COX4-1 reverses chemoresistance to TMZ [12, 17], supporting a close correlation between acquired chemoresistance and changes in cellular metabolic machinery at the level of the mitochondrion. Thus, a mechanism to therapeutically target CcO activity may provide substantial benefit to patients with GBM.

Chlorpromazine (CPZ) is one of the oldest drugs developed as an antipsychotic agent [29–31], but interest in this drug has been revived as the anti-cancer activity of CPZ has been demonstrated experimentally in many cancers, including glioma [32–38]. However, the specificity of CPZ as an agent against chemoresistant glioma has never been tested. Remarkably, it was reported > 50 years ago that CPZ affects mitochondrial function by blocking CcO activity [39, 40], but the therapeutic relevance of mitochondrial activity, and thus of this finding, was not apparent at the time. Therefore, the mechanisms by which CPZ blocks CcO activity as well as the therapeutic potential of this effect remain unknown. To obtain insight into the action of CPZ in the regulation of CcO and the anti-glioma properties of CPZ against TMZ-resistant cells, we analyzed CcO activity, cell cycle control, and long-term clonogenic survival in glioma cells after CPZ treatment. We found that CPZ specifically inhibits CcO activity in chemoresistant glioma cells, including glioma stem cells (GSCs) derived from patient xenolines.

## RESULTS

### CPZ inhibits proliferation in chemoresistant glioma and GSCs

**Figure 1:**
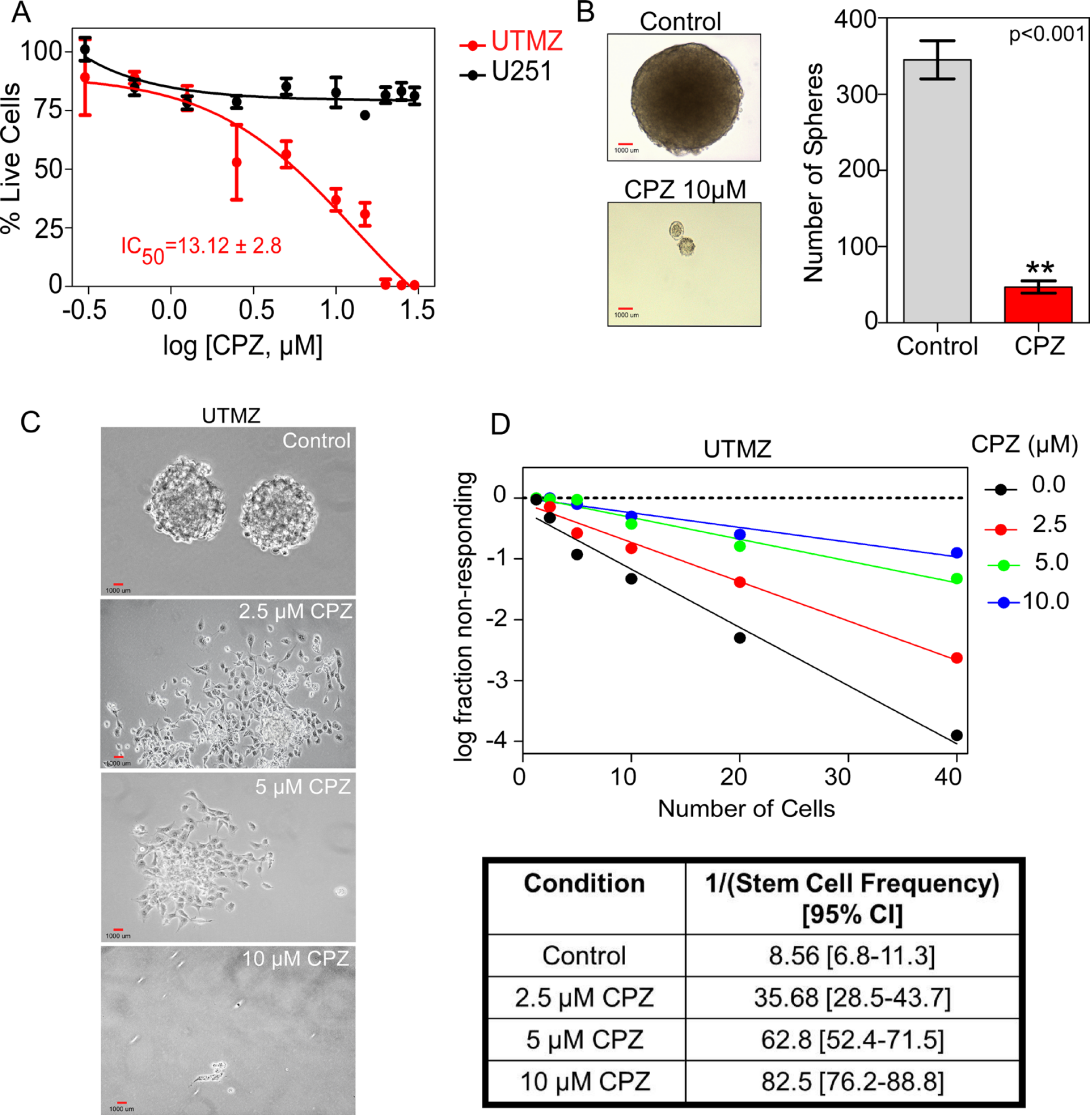
Effect of CPZ on proliferation of TMZ-resistant cells (**A**) Effect of CPZ on TMZ-sensitive U251 and TMZ-resistant UTMZ glioma cell proliferation. Cells were treated with CPZ at the indicated concentrations. (**B**) Anchorage-independent growth, assessed by colony formation of UTMZ cells in semisolid medium. Cells were grown on soft agar plates for 3 weeks before colonies were visualized microscopically. Left panel: Representative micrographs of vehicle-treated (top) and CPZ-treated cells (bottom). Right panel: Quantification of colony formation. Colonies were counted in a blinded fashion. (**C**) Representative micrographs from *in vitro* limiting dilution assays with GSCs treated with PBS or CPZ at the indicated concentrations. (**D**) Quantification of GSCs in the respective assays in (C). Results represent the average from two independent experiments.

Because we hypothesized that CPZ has an anti-proliferative effect on chemoresistant glioma, the effect of CPZ on cell survival and growth was examined. We first used the TMZ-sensitive human glioma cell line U251 and a U251-derived TMZ-resistant cell line (UTMZ) previously described [11]. Treatment with CPZ induced inhibition of cell proliferation in UTMZ cells with an IC_50_ of 13.12 ± 2.8 μM. In contrast, CPZ did not affect cell proliferation of TMZ-sensitive U251 cells at concentrations up to 30 μM and with treatment times up to 96 h (Figure 1A). CPZ also significantly inhibited anchorage-independent growth of UTMZ cells (*p* < 0.001) in soft agar growth assays (Figure 1B). Because CPZ blocked cell proliferation specifically in chemoresistant glioma cells, we investigated whether CPZ blocks cell proliferation in the proportion of TMZ-resistant cells that have GSC properties. As illustrated in Figure 1C, when cultured in serum-free culture medium supplemented with epidermal growth factor (EGF) and basic fibroblast growth factor (bFGF), TMZ-resistant UTMZ cells formed neurospheres ranging from 0.1 to 1 mm in diameter. However, when UTMZ cells were cultured in the presence of CPZ, smaller and fewer neurospheres developed, ranging from 2.5 to 10 μm in diameter. When cells were plated in an *in vitro* limiting dilution assay, CPZ also inhibited the formation of tumor neurospheres in a dose-dependent manner (Figure 1D).

### CPZ inhibits CcO activity

**Figure 2:**
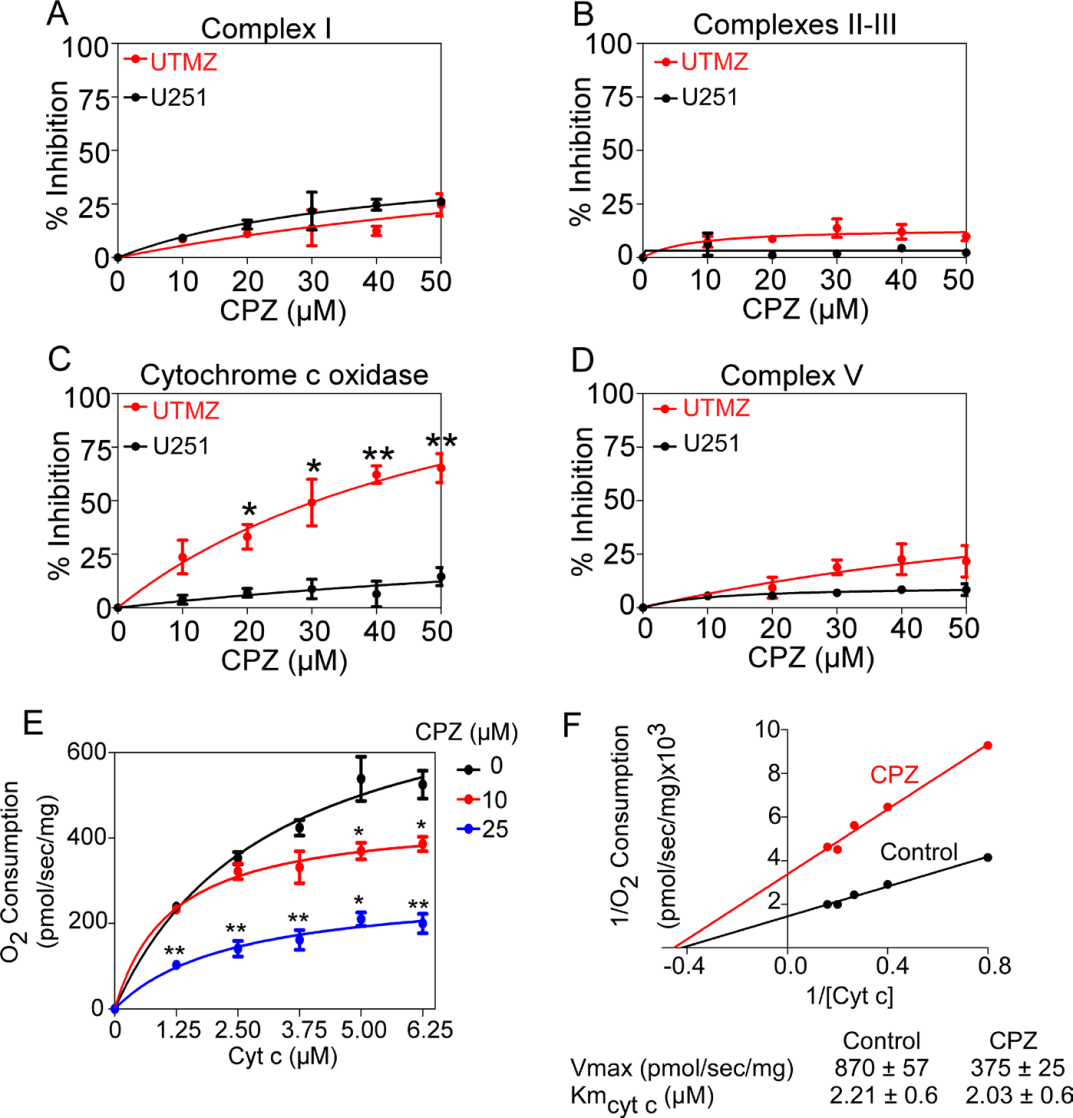
Effects of CPZ on mitochondrial complexes (**A**–**D**) CPZ was tested on mitochondrial extracts from TMZ-sensitive U251 and TMZ-resistant UTMZ glioma cells to determine the effects on the activity of complex I (A), II-III (B), CcO (complex IV) (C), and complex V (D) of the mitochondrial transport chain. Graphs represent the activity level of each complex in the presence of PBS (control) or CPZ (up to 50 μM). The results are averages from triplicate determinations from two independent experiments. (**E**) Representative Michaelis-Menten graph depicting the inhibition of cyt c activity by CPZ. (**F**) Representative Lineweaver–Burk double-reciprocal plots indicating a non-competitive inhibition of cyt c, with a 50% decrease in Vmax at 2 μM CPZ. *p < 0.05; **p < 0.01; and ***p < 0.001.

CPZ has been reported to target mitochondrial function [39, 40], thus we tested whether CPZ targets the mitochondrial ETC complexes. The activities of complexes I, II–III, IV (CcO) and V (ATP synthase) were measured in mitochondrial extracts from TMZ-sensitive U251 and TMZ-resistant UTMZ cells in the presence of differing CPZ concentrations (Figure 2). Although CPZ did not affect complexes I, II–III, or V (Figure 2A, 2B and 2D), it significantly decreased CcO activity in a dose-dependent manner (Figure 2C) specifically in UTMZ cells. We next investigated the kinetic mechanism of CPZ inhibition of CcO. CPZ lowered the Vmax (870 ± 57 to 375 ± 24 pmol/sec/mg) but not the Km for cyt c. Figure 2E shows the representative Michaelis-Menten graph, and Figure 2F shows the representative Lineweaver–Burk double-reciprocal plots indicating a non-competitive inhibition of cyt c, with a 50% decrease in Vmax at 2 μM CPZ.

**Figure 3:**
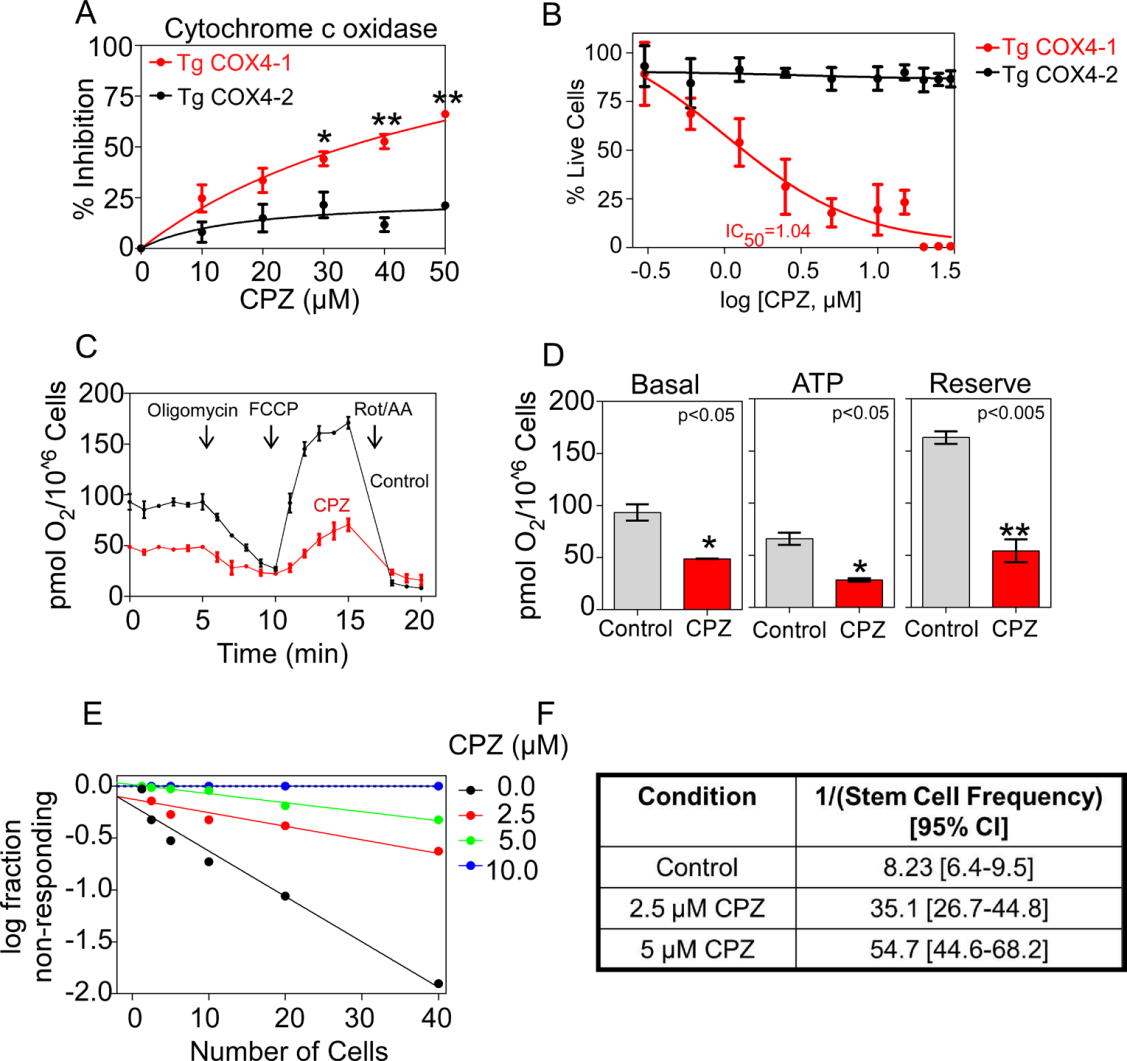
Effects of CPZ on CcO expressing COX4-1 or COX4-2 isoform (**A**) The effect of CPZ on CcO activity was tested in mitochondrial extracts from cells with CcO expressing COX4-1 (TgCOX4-1) or COX4-2 (TgCOX4-2). Graphs represent the level at which CcO activity is inhibited in the presence of PBS (control) or CPZ (up to 50 μM). The results are averages from triplicate determinations from two independent experiments. (**B**) Effect of CPZ on the proliferation of cells with CcO expressing COX4-1 or COX4-2. (**C**) Bioenergetic profile of TgCOX4-1 intact cells in the presence of PBS (control) or CPZ (5 μM). Oxygen consumption was measured using sequential injection of oligomycin (2 μg/ml), FCCP (1 μM), and antimycin A (2.5 μM). (**D**) Basal respiration, ATP-linked respiration and reserve respiratory capacity are shown. Results represent the means ± SD of 3 independent experiments. (**E**) and (**F**) *In vitro* limiting dilution assays and quantification of GSCs from cells with CcO expressing COX4-1 and treated with CPZ at the concentrations indicated. Results are the average from two independent experiments. *p < 0.05; **p < 0.01; and ***p < 0.001.

Because we previously demonstrated that the expression of COX4-1, rather than COX4-2, is in part responsible for the expansion of GSCs [11], the cells implicated in tumor recurrence and resistance to therapy in patients with glioblastoma, we tested the effect of CPZ in U251 glioma cells transfected with FLAG-epitope-tagged COX4-1 (U251-TgCOX4-1) or FLAG-epitope-tagged COX4-2 (U251-TgCOX4-2). U251 cells express the COX4-2 isoform, thus the vectors were transfected into U251 cells stably depleted of endogenous COX4-2 [11]. As illustrated in Figure 3, CPZ inhibited CcO activity and decreased the proliferation of cells that expressed the COX4-1 isoform, with an IC_50_ of 1.04 μM (Figure 3B).

To provide evidence that cell treatment with CPZ inhibits O_2_ consumption, we investigated the cellular bioenergetic response to CPZ by high-resolution respirometry. A comparison of different parameters in COX4-1– and COX4-2–expressing glioma cells is provided in Figure 3. Under basal conditions, CPZ-treated COX4-1 glioma cells had markedly lower basal mitochondrial respiration than untreated COX4-1–expressing cells had (Figure 3C, 3D). This mitochondrial respiration is composed of two components: the O_2_ consumption related to ATP synthesis and the O_2_ consumption due to the proton leak across the inner mitochondrial membrane. The addition of oligomycin, an ATP synthase inhibitor, allowed the differentiation of these two parameters. While there was no significant difference in proton leak, the ATP-linked respiration was significantly reduced in COX4-1 cells pretreated with CPZ (Figure 3C, 3D). The addition of the uncoupler FCCP allowed the determination of the potential maximal respiratory capacity of the cells. COX4-1–expressing cells pretreated with CPZ had a markedly lower maximum respiratory rate than untreated COX4-1-expressing cells had (Figure 3C, 3D). Compared with COX4-1–expressing glioma cells, COX4-2–expressing glioma cells have a reduced respiratory capacity. Notably, CPZ did not induce significant differences in basal, ATP-linked, or maximal respiration in glioma cells expressing COX4-2, suggesting that the lack of COX4-1 isoform makes the cells insensitive to CPZ (data not shown).

When cells overexpressing COX4-1 were plated in an *in vitro* limiting dilution assay, CPZ inhibited the formation of tumor neurospheres and the frequency of self-renewing cells in a dose-dependent manner (Figure 3E, 3F). The detection of CPZ-mediated inhibition of CcO activity only when the COX4-1 subunit was expressed suggests that COX4 isoform-specific differences in CcO structure may control the effects of CPZ on CcO.

**Figure 4:**
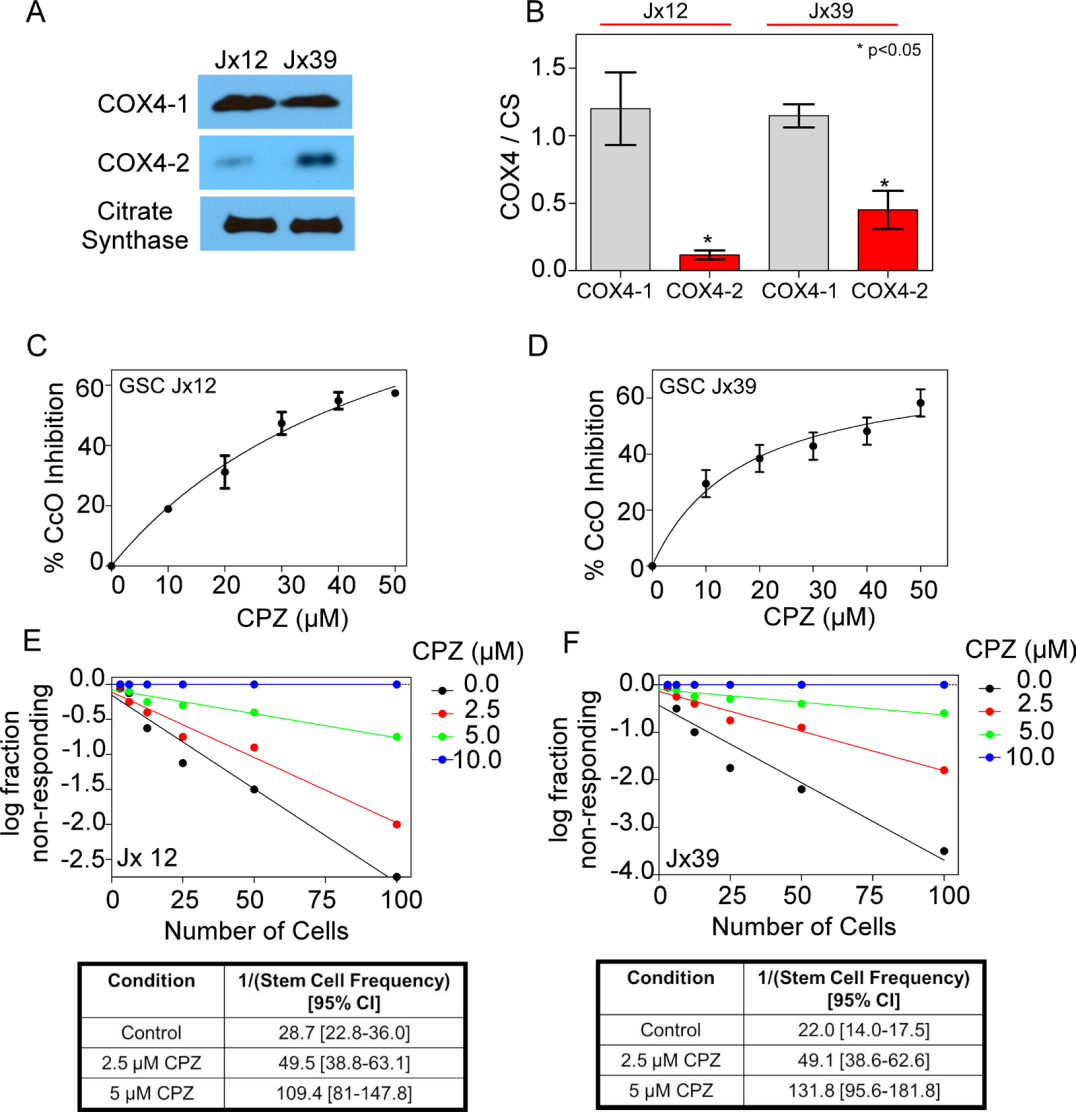
Effects of CPZ on GSCs from human xenografts (**A**) Representative Western blot showing the relative expression levels of endogenous COX4-1 and COX4-2 isoforms isolated from mitochondrial fractions of GSCs from J × 12 and J × 39 xenolines. (**B**) Quantitative analysis of expression levels of COX4-1 and COX4-2 isoforms relative to citrate synthase (CS; loading control). Bars represent the average from duplicate determinations (*p < 0.05). C, D. The effect of CPZ on the activity of CcO (complex IV), specifically, was tested in mitochondrial extracts from J × 12 (**C**) and J × 39 (**D**) xenolines. Graphs represent the level at which CcO activity is inhibited in the presence of 0 μM CPZ (control) or up to 50 μM CPZ. The results are averages from triplicate determinations from two independent experiments. (**E**, **F**) *In vitro* limiting dilution assays (top panels) and quantification of GSCs (bottom panels) from J × 12 (E) and J × 39 (F) xenolines treated with CPZ at the concentrations indicated. Results represent the average from two independent experiments.

We next investigated the effect of CPZ on mitochondrial extracts from GSCs derived from J × 12 and J × 39 human tissue. Western blot analysis of the COX4 subunits showed that both human GSC types expressed significantly higher levels of COX4-1 than of COX4-2 (Figure 4A and 4B). CPZ also decreased CcO activity in GSCs derived from both J × 12 and J × 39 xenolines (Figure 4C and 4D). Similarly, CPZ significantly decreased the stem cell frequency and the frequency of self-renewing cells in a dose-dependent manner in both xenolines (Figure 4E and 4F).

### CPZ induces cell cycle arrest in TMZ-resistant glioma cells

To evaluate the inhibitory effect of CPZ on cell proliferation, we measured cell-cycle progression using flow cytometry. TMZ-sensitive U251 and TMZ-resistant UTMZ cells were treated with 10 or 20 μM of CPZ, and the percentage of cells in each stage of the cell cycle was determined 24 h after treatment, as described in the materials and methods section.

**Figure 5:**
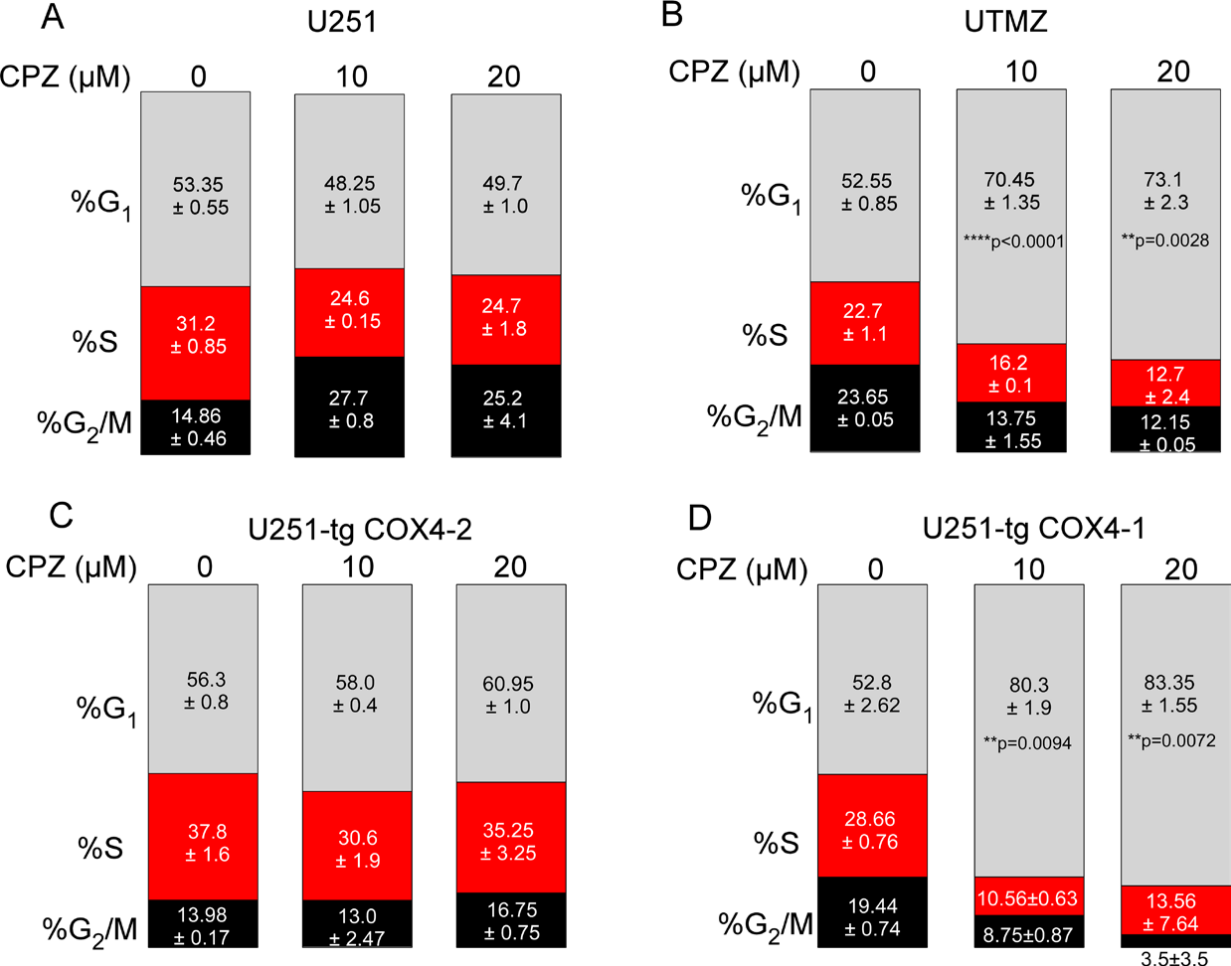
Effect of CPZ on cell cycle (**A**–**D**) Average distributions of cells in G1, S, and G2/M phases for TMZ-sensitive U251 glioma cells (A), TMZ-resistant UTMZ glioma cells (B), and U251 glioma cells with CcO expressing COX4-2 (C) or COX4-1 (D). Cells were exposed to 0, 10, or 20 μM CPZ for 24 h. Results represent the average from two independent experiments.

In agreement with the results of the proliferation studies (Figure 1A), CPZ treatment of U251 cells did not affect the percentage of cells in the G1 phase (Figure 5A). However, CPZ treatment of UTMZ cells led to G1 phase accumulation that reached 70.4% (*p* < 0.0001) and 73.1% (*p* = 0.0028) after treatment with 10 and 20 μM CPZ, respectively, whereas only 52.6% of control vehicle-treated UTMZ cells were in G1 (Figure 5B). Concomitantly, 12.1% of UTMZ cells in the 20 μM CPZ group were in the S phase fraction compared with 22.7% of the cells in the control group. Similarly, 12.2% of UTMZ cells in the 20 μM CPZ group were in the G2/M fraction compared with 23.6% of cells in the control group. These results indicate that CPZ causes inhibition of cell proliferation in TMZ-resistant cells by G1 cell cycle arrest.

Because CPZ blocks CcO activity and cell proliferation when COX4-1 is expressed, we next compared the effect of CPZ on cell cycle progression in glioma cells transfected with COX4-1 (U251-TgCOX4-1) or with COX4-2 (U251-TgCOX4-2). As shown in Figure 5C and 5D, CPZ triggered cell cycle arrest only when cells expressed COX4-1. Indeed, the percentage of glioma cells transfected with TgCOX4-1 accumulated in the G1 phase increased from 52.8% in control-treated cells to 83.5% in cells treated with 20 μM CPZ (*p* = 0.0072).

### CPZ inhibits CcO activity from bovine heart

**Figure 6:**
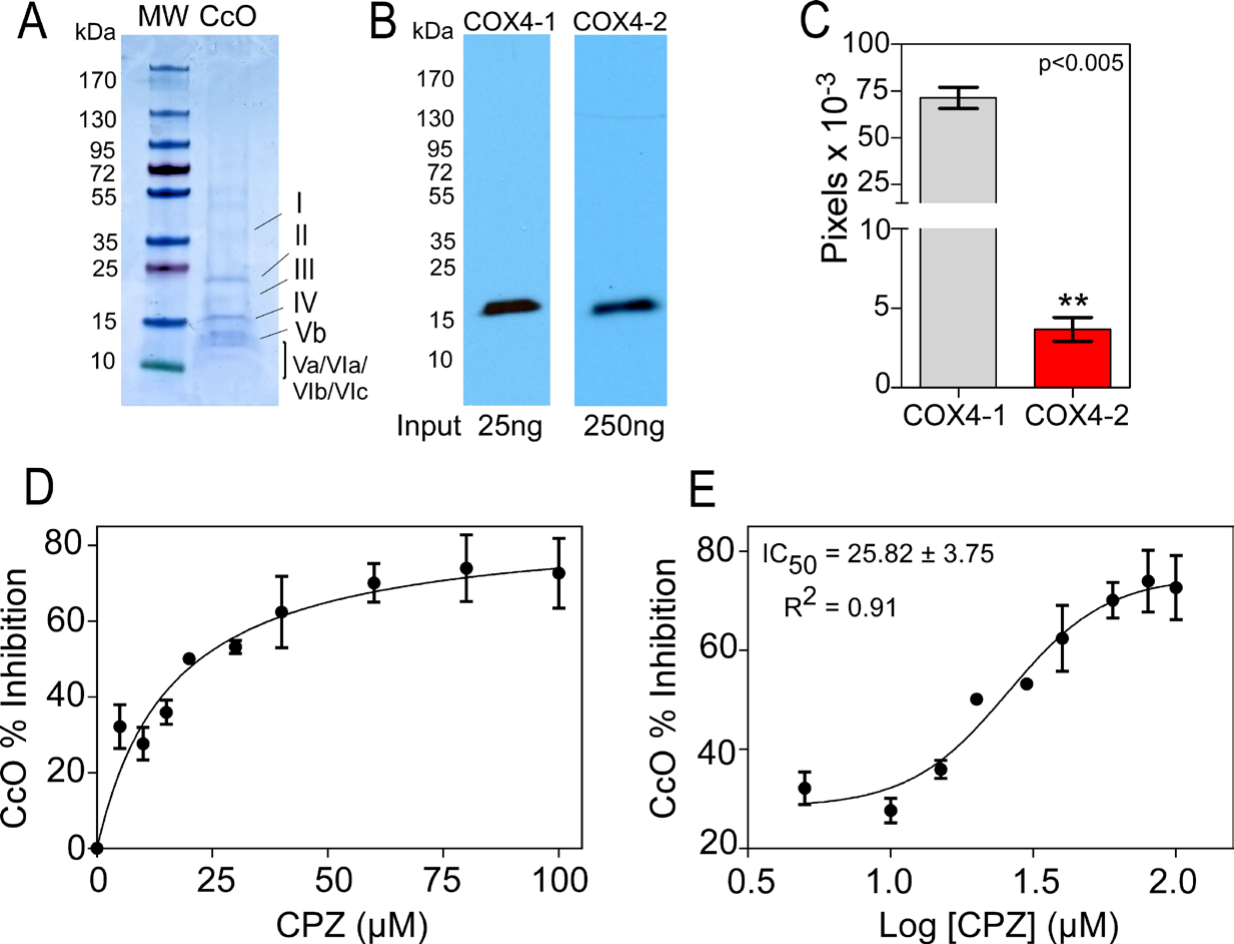
Effect of CPZ on CcO purified from bovine heart (**A**) Representative SDS-PAGE stained with Coomassie blue. Lane 1, protein ladder. Lane 2, purified CcO from bovine heart (5 μg). (**B**) Representative Western blot showing the expression of COX4-1 and COX4-2 isoforms in CcO purified from bovine heart. (**C**) Quantitative analysis of the relative expression levels of COX4-1 and COX4-2 isoforms in CcO from bovine heart. Bars represent the average from duplicate determinations. (**D**) Inhibition of CcO activity in the presence of 0 μM CPZ (control) and CPZ up to 100 μM. (**E**) Determination of IC_50_. The results are averages from triplicate determinations from two independent experiments.

Because CPZ was tested using mitochondrial extracts from glioma cells, we assessed the possibility that the resulting CcO inhibition was an indirect effect due to binding to other mitochondrial components by evaluating the effect of CPZ on CcO purified from bovine heart. When evaluated by SDS-PAGE, the purified CcO enzymes produced multiple bands of apparent molecular mass ranging from 12 to 45 kDa (Figure 6A). Western blot analysis demonstrated that COX4-1 was the most abundant COX4 isoform expressed (Figure 6B), with expression levels about 20-fold higher than the levels of COX4-2 (Figure 6C). CPZ significantly decreased the activity of purified CcO in a dose-dependent manner (Figure 6D), with an IC_50_ of 25.82 ± 3.75 μM (Figure 6E). These results demonstrate a direct interaction of CPZ with CcO.

### CPZ prolongs the survival time of mice bearing TMZ-resistant glioma cells

**Figure 7:**
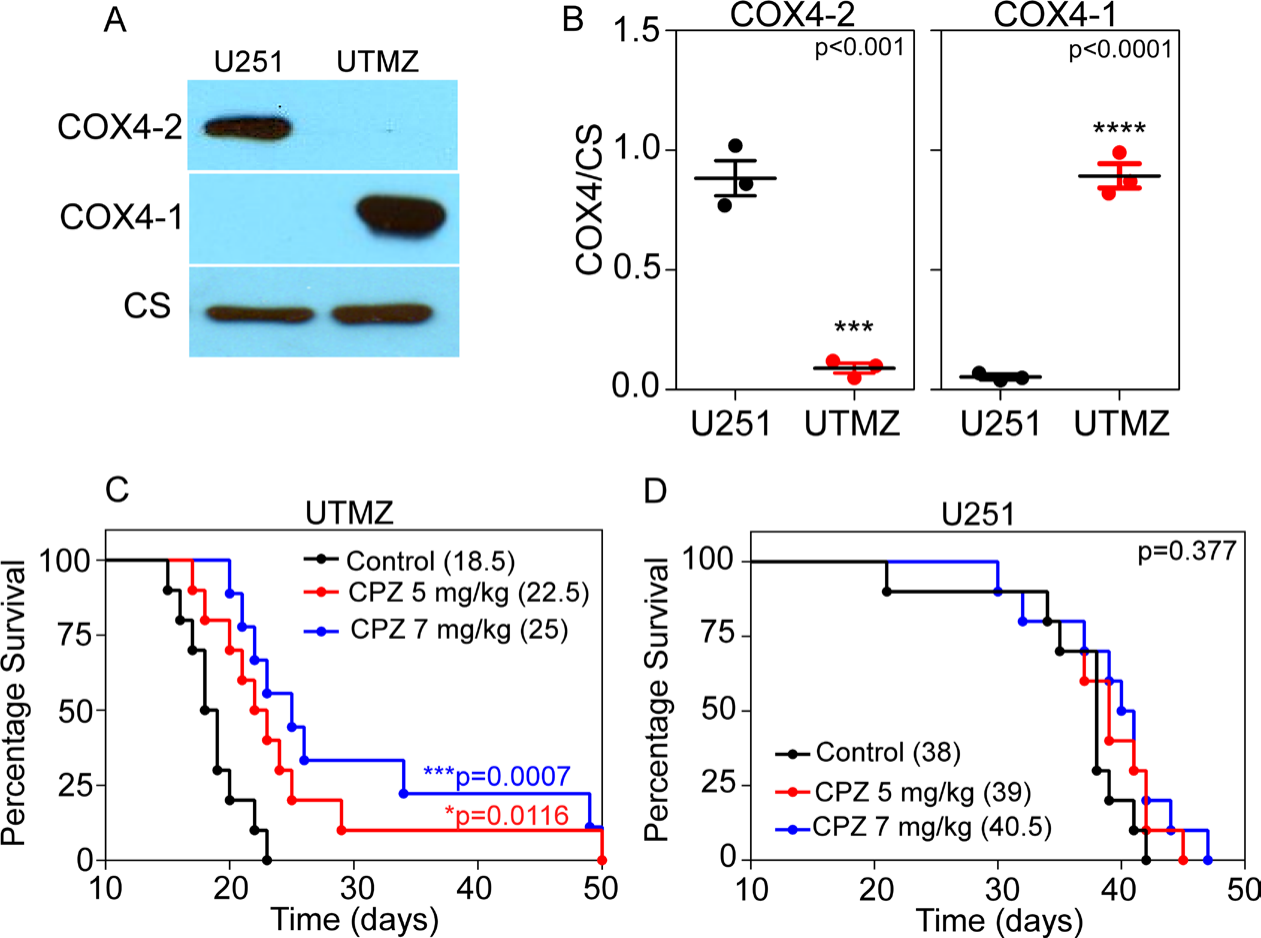
Survival data for CPZ-treated and untreated glioma-bearing mice (**A**) Representative Western blot showing the expression of COX4-1 and COX4-2 isoforms in CcO from TMZ-resistant UTMZ or TMZ-sensitive U251 cells. (**B**) Quantitative analysis of the relative expression levels of COX4-1 and COX4-2 isoforms in CcO from U251 and UTMZ cells. Results represent the average from duplicate determinations. (**C**, **D**) TMZ-resistant UTMZ (C) or TMZ-sensitive U251 (D) glioma-bearing mice were treated with CPZ (5 or 7 mg/kg) or with saline (vehicle) as a control (*n* = 10 mice/group). In mice bearing UTMZ tumors, CPZ treatment significantly improved survival (***p = 0.0007 versus saline). In mice bearing U251 tumors, there was no significant difference in survival between CPZ-treated and saline-treated controls (*p* = 0.377).

To study whether CPZ is effective in an orthotopic mouse models, we stereotactically injected TMZ-sensitive U251 and TMZ-resistant UTMZ tumor cells into the right caudate/putamen of nude mice. Western blot analysis demonstrated that COX4-1 was the most abundant COX4 isoform expressed in the TMZ-resistant cell line (UTMZ) (Figure 7A), with expression levels about 10-fold higher than the levels of COX4-2 (Figure 7B). In contrast, the TMZ-sensitive cell line (U251) expressed mostly the COX4-2 isoform. After tumor inoculation, the mice were randomly allocated into three groups of 10 mice each and treated with saline or CPZ at 5 or 7 mg/kg, injected intraperitoneally three times a week for 2 weeks and 5 days after tumor implantation. Median survival of mice was 18.5 days in the saline group and increased to 22.5 days in the 5 mg/kg CPZ group (*p* = 0.01) and 25.0 days in the 7 mg/kg CPZ group (*p* = 0.0007) (Figure 7C). In contrast, CPZ treatment of mice bearing U251 glioma cells provided no benefit in median survival as determined by log-rank test (Figure 7D). No differences in behavior or weight were observed between saline-treated and CPZ-treated mice (data not shown).

### CPZ likely binds to a hydrophobic pocket formed by COX4 and COX1

**Figure 8:**
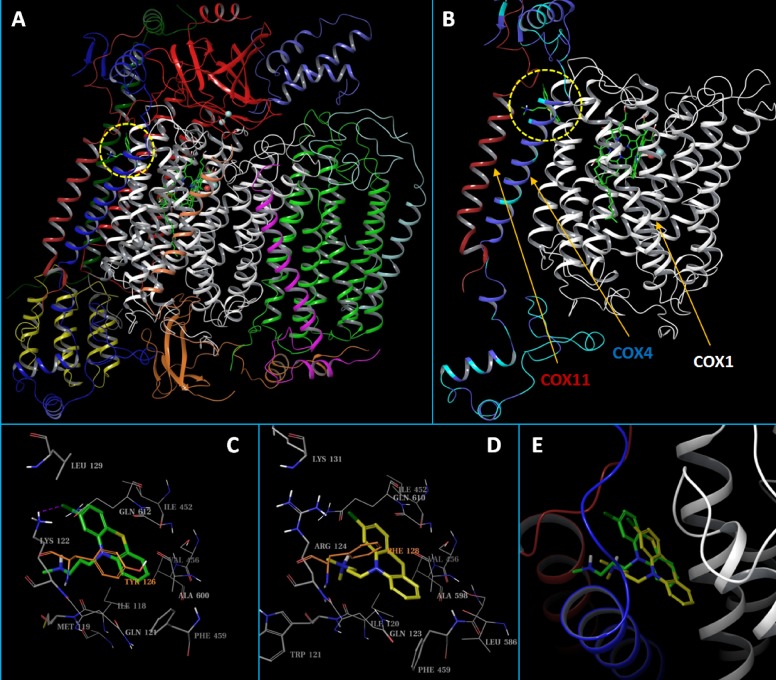
Structural presentation of the predicted binding modes of CPZ to human CcO (**A**) Carton presentation of the CcO complex model with each of its 13 subunits shown in differently colored ribbons. The predicted binding site, siteA, was circled in a dashed yellow line. (**B**) CcO subunits that are close to siteA. The HEME molecule and the docked CPZ molecule are shown in solid sticks. COX1 and COX11 are shown in white and red ribbons, respectively. For the COX4 ribbon, residues that are the same between COX4-1 and COX4-2 were colored in blue, while residues that are different are colored in cyan. (**C** and **D**) Close up view of the binding site interactions of the docked CPZ in the COX4-1 and the COX4-2 models, respectively. The CPZ molecules are shown in solid sticks and colored in green and yellow, respectively. Binding site residues are shown in gray lines. The key and non-conserved residues are shown in thin sticks, or colored in orange. (**E**) Overlaid docked CPZ molecules from the COX4-1 and COX4-2 models. CPZs and CcO ribbons are colored the same as above.

To explore the potential mechanism of CPZ/CcO binding, we constructed human CcO homology models and conducted structural analysis and molecular docking studies. CcO is a large protein complex with 13 different subunits (Figure 8A). Homology models of human CcO were built based on a crystal structure of mouse CcO. The residues in the mouse CcO COX4 subunit are 82% identical to and 96% homologous to the residues of human COX4-1 and 46% identical to and 64% homologous to the residues in human COX4-2. As a comparison, the residues in human COX4-1 are 45% identical to and 58% homologous to residues in human COX4-2. Modeling showed that the COX4 subunit adopts a structure with a long helical middle fragment and two partly folded but mostly unstructured terminal ends (Figure 8B). Our structural mapping analysis indicated that the COX4 alone does not provide proper sites for ligand binding, but the CcO complex contains several potential binding sites that involve COX4 residues. Analysis of CPZ docking revealed one site as the most likely binding site for CPZ (Figure 8A and 8B). This identified site, which we named siteA, is mainly a hydrophobic pocket and is formed by residues from the central helical region of COX4 and two other transmembrane helices of COX1 that are close to the HEME binding site. SiteA has an excellent SiteScore of 0.92 (a SiteScore value of 0.80 has been found to accurately distinguish between drug-binding and non-drug-binding sites [41]) and consists of residues that differ between COX4-1 and COX4-2, including Leu129/Lys131, Lys122/Arg124, Met119/Trp121, and Tyr126/Phe128 from COX4-1/COX4-2, respectively. The docked CPZ-COX4-1 model score (-6.0 kcal/mol) was significantly better than the score of the docked CPZ-COX4-2 model (-5.3 kcal/mol), suggesting CPZ binds more tightly to CcO with COX4-1 than to CcO with COX4-2. Such a result can be well explained by the different residues at siteA. The hydrophobic Leu129 makes the siteA of COX4-1 a partly closed pocket that is a more comfortable environment for CPZ binding; as a result, CPZ is able to form an H-bond with Lys122 and π-π stacking interaction with the phenol ring of Tyr126 (Figure 8C). In contrast, the highly polar Lys131 of COX4-2 opens up the siteA, which makes the mainly hydrophobic CPZ molecule move toward the inner part of the pocket, and the relatively smaller sidechain of Phe128 (compared with Tyr126 of COX4-1) further permits such a movement. Additionally, the flat amide end of Arg124 (compared with Lys122 of COX4-1) impedes the formation of the H-bond, thus both the H-bond and the π-π stacking interaction are lost in the docked COX4-2 model (Figure 8D). Interestingly, in the CcO complex, COX11 locates very close to siteA, and in the docked COX4-1 model, the dimethylamine end of CPZ overlapped with residues of COX11 (Figure 8E). Therefore, CPZ binding would likely generate a steric hindrance that blocks the interactions between COX11 and the rest of the CcO complex, while the deeply buried CPZ identified in the COX4-2 model would not have such an effect (Figure 8E).

## DISCUSSION

The Warburg effect, in which cells exhibit increases in glucose uptake, glycolytic capacity, and lactate production and the absence of respiration despite a high oxygen concentration, is often detected in tumor cells [3]. However, we and other groups have identified several glioma cell lines that are highly dependent on the mitochondrial OxPhos pathway to produce ATP [42–47]. Furthermore, we reported that a subclass of glioma cells that utilize glycolysis preferentially can also switch from aerobic glycolysis to OxPhos under limiting glucose conditions, as has been observed in cervical cancer cells, breast carcinoma cells, hepatoma cells, and pancreatic cancer cells as well [45–47]. Furthermore, a recent study demonstrated that mitochondrial ROS promote glioma progression [48]. Thus, targeting mitochondria could provide therapeutic opportunities.

In a retrospective clinical trial in patients with newly diagnosed primary GBM, we identified a subset of patients (25–30% of the study population) with an extremely low overall survival (6 months) and high resistance to therapy [23]. Tumors in this population are characterized by less heterogeneity, OxPhos-dependent metabolism, elevated CcO activity, and enrichment in GSCs. Similar results from another research group identified patients with GBM characterized by low overall survival and tumors with upregulated expression levels of metabolic enzymes, including CcO [49]. We have also demonstrated that during tumor regrowth (recurrence), a switch from glycolytic to OxPhos metabolism may occur with a concomitant increase of CcO activity [12, 17]. Additionally, we showed that genetic and pharmacologic inhibition of CcO activity suppresses tumor growth and depletes GSCs in gliomas, providing a strategic opportunity to improve therapeutic outcomes in patients with GBM [12, 17, 50]. With respect to this concept, we recently identified and characterized a novel small molecule inhibitor of CcO (ADDA 5) [50]. ADDA 5 inhibits the proliferation of glioma cells, without toxicity against non-cancer cells, and treatment with ADDA 5 significantly inhibits tumor growth in flank xenograft mouse models. Importantly, ADDA 5 inhibits CcO activity and blocks cell proliferation and neurosphere formation of GSCs [50].

However, while ADDA 5 is a promising compound for the treatment of GBM, clinical translation of this compound may take years. The repositioning of existing Food and Drug Administration (FDA)-approved drugs can bypass or shorten critical steps of drug development, such as chemical optimization and toxicology testing, thereby resulting in a shorter time frame for clinical translation and patient benefits. We propose that FDA-approved agents that cross the blood brain barrier, such as CPZ, can be repurposed for use in the treatment of chemoresistant GBM.

Although CPZ-mediated blockade of CcO activity was described > 50 years ago [39], the effects of CPZ on mitochondrial ETC complexes specifically in glioma cells were not examined. Our study of these effects revealed that CPZ does not affect complexes I, II–III, or V. Notably, CPZ did significantly decrease CcO activity, but only in TMZ-resistant glioma cells. We previously showed that acquisition of TMZ-resistance is associated with a switch in the regulatory subunit of CcO such that the COX4-1 isoform, rather than the COX4-2 isoform, is mainly expressed [12]. To determine if the expression of COX4-1 underlies the specificity of CPZ for TMZ-resistant cells, we compared the effects of CPZ on CcO from glioma cells expressing COX4-1 and from cells expressing COX4-2 [11]. CPZ inhibited CcO activity only when the COX4-1 subunit was expressed, suggesting that the COX4 isoform-specific differences in CcO structure control the interaction with CPZ.

Analysis of the mechanism of inhibition showed that CPZ is a non-competitive inhibitor of CcO with respect to the cyt c substrate, providing strong evidence that CPZ binds to an allosteric site rather than to the active site of cyt c. Of note, it was previously shown that CPZ blocks CcO activity by a competitive mechanism that blocks the interaction with cyt c [39, 40]. The discrepancy with our results may be due to cell type-specific responses, differences in normal and cancer cells, or variations in experimental conditions. Specifically, the results reported by Dawkins et al. [39] reflect the effect of CPZ on the activity of CcO from normal liver mitochondria, whereas our study characterized the effect of CPZ on CcO from human brain cancer cells. Furthermore, the concentrations of CPZ which they found necessary to inhibit CcO activity were significantly higher than the one reported here (150–200 μM vs 10–30 μM), which may be a consequence of the high concentrations of cyt c tested in their system (30–200 μM).

Our computer modeling studies suggested that CPZ binds to a hydrophobic pocket formed by residues from the central helical region of COX4 and two other transmembrane helices of COX1 that are close to the HEME binding site. Our docking studies also suggested that CPZ binds more favorably to CcO with COX4-1 than with COX4-2, and may affect CcO function through direct interactions with COX1 residues that are close to the HEME center, by preventing the recruitment of COX11, or through a combination of these mechanisms.

*In vitro*, CPZ inhibited cell proliferation and anchorage-independent growth in chemoresistant but not chemosensitive glioma cells. CPZ also inhibited cell proliferation of GSCs. These data suggest that CPZ would be effective in minimizing recurrence that may result from the proliferation and differentiation of GSCs.

Our results showed that the growth inhibition is achieved at a concentration of CPZ between 1 and 15 μM. Since we evaluated different cellular processes (attached cell growth and anchorage-independent cell growth), it is absolutely possible that both processes are differentially affected by CPZ. Indeed, the concentration necessary to block CcO activity *in vitro* is significantly higher (IC_50_ = 30 μM). It is possible that the mitochondrial membrane potential can increase uptake and/or prolong retention of CPZ within the mitochondria, reducing the IC_50_ in intact cells.

Notably, CPZ treatment substantially increased the survival of mice bearing intracranial TMZ-resistant glioma cells, without any noticeable adverse effects such as change in animal weight or behavior, implying a potential therapeutic application for CPZ.

CcO is widely expressed in mitochondria of eukaryotes, which raises concern about systemic inhibition of CcO. However, mitochondria have emerged recently as effective targets for novel anti-cancer drugs with high specificity for cancer cells [50–63]. Indeed, we have identified the molecular mechanisms that permit the malignant cell-selectivity of inhibitors of CcO. Normal mammalian cells have a relatively large excess (3- to 10-fold) of CcO activity compared with that in primary and recurrent GBM [64–69]. In normal brain cells, therefore, the CcO activity could be decreased by approximately 70% before major changes in mitochondrial respiration and ATP synthesis occur [64, 65], whereas a 7–22% decrease in CcO activity is sufficient to promote the alteration of energy homeostasis in malignant cells [12, 69]. Thus, the higher CcO activity and threshold in respiration in normal cells offers a window for the therapeutic use of CcO-specific inhibitors, since the doses necessary to effectively decrease CcO activity in malignant cells should not affect the overall capability of CcO to maintain energy homeostasis in normal cells.

Furthermore, we demonstrated that CPZ blocks proliferation of chemoresistant gliomas. Interestingly, cell cycle analyses showed that the decreased proliferative activity is due to cell cycle arrest, rather than increased apoptosis. Indeed, we detected few apoptotic cells and the number did not significantly increase throughout CPZ treatment (data not shown). CPZ has also been shown to inhibit cell-cycle progression in rat glioma C6 cells by inducing p21Waf/Cip1/Egr1 expression [36] and to induce autophagic cell death by inhibiting the AKT/mTOR pathway in human glioma U87-MG cells [37]. In addition, CPZ enhanced therapeutic efficacy in tamoxifen-resistant breast cancer cells [38] and in colorectal cancer [34], suggesting this drug as a potential agent for improving the efficacy of cancer chemotherapy.

In conclusion, our study provides new insight into the repositioning of CPZ for the treatment of chemoresistant gliomas. Future studies should investigate the potential interactions between CPZ and other conventional anti-GBM therapies.

## MATERIALS AND METHODS

### Cell culture

TMZ-sensitive U251 cells and TMZ-resistant cells derived from U251 cells (UTMZ) were grown in DMEM F-12 medium plus l-glutamine supplemented with 7% heat-inactivated FBS, penicillin, and streptomycin as we previously described [11, 12, 17, 42, 50, 55, 70, 71]. The resistant cell line was obtained by progressive adaptation of the parental sensitive cells (U251) to increasing concentrations of TMZ [12]. FLAG-epitope-tagged COX4-2 and COX4-1 were generated and transfected into U251 cells stably depleted of the endogenously expressed COX4-2, as previously described [11].

Cell lines are grown continuously up to 10 passages, and then we start a new culture from frozen seed stocks. Cell lines are regularly tested for mycoplasma contamination using the Universal Mycoplasma Detection kit (ATCC^®^ 30-1012KTM) and authenticated by the ATCC authentication service utilizing short tandem repeat (STR) profiling.

### Cell proliferation assay

For cell proliferation, glioma cells were seeded into 24-well plates (3 × 10^4^ cells/well), and cell number was counted every 24 h for 4 days as previously described [11].

### Soft agar growth assay

A bottom layer of 0.4% agarose and DMEM/F12 with 10% FBS was poured and allowed to solidify. Additional agarose was allowed to reach 42°C and then 7.5 × 10^3^ tumor cells were added to the agarose/media solution and poured onto the bottom layer. Appropriate concentrations of CPZ were added to both agarose/media layers. Cells were incubated at 37°C for 4 weeks to form colonies, followed by staining with 0.005% crystal violet. The colonies were imaged and quantified using the Gel Dock imager and Quantity One software (BioRad).

### *In vitro* limiting dilution assay

*In vitro* dilution assays were performed as previously described [11]. Briefly, single-cell suspensions were plated at 1, 2, 5, 10, 20, and 40 cells per well in 96-well plates in neurobasal medium containing EGF and FGF. Ten days after plating, the number of neurospheres in each well and the percentage of positive wells were quantified by manual counting. Extreme limiting dilution assay analyses were performed on the data as previously described [11, 44, 45].

### Primary GBM xenograft lines

The establishment and maintenance of the Mayo GBM xenograft lines J × 12 and J × 39 has been described [12, 17, 72–76]. J × 12 and J × 39 are classical subtype patient-derived GBM xenograft cell lines (xenolines) that were established in immunocompromised athymic nude mice from surgical resection waste specimens obtained from consented patients undergoing surgical therapy for primary GBM. All animal studies were approved by the UAB Institutional Animal Care and Use Committee at the University of Alabama at Birmingham.

### Intracranial tumors

All surgical and experimental procedures and animal care were performed in accordance and compliance with the policies approved by the University of Alabama at Birmingham Institutional Animal Care and Use Committee (APN 131209529) as we previously described (11). Briefly, intracranial gliomas were generated using 3 × 10^5^ U251 or UTMZ human glioma cells suspended in 5% methylcellulose in serum-free medium. The cells were drawn into a 250-μl Hamilton gas-tight syringe mounted in a Chaney repeating dispenser and fitted with a 30G ½-inch needle with a calibrated depth of 2.5 mm from the middle of the bevel opening. Under an operating microscope, the fascia on the skull of the anesthetized mouse was scraped off and a 0.5-mm burr hole was made 2 mm to the right of the midline suture and 1 mm caudal to the coronal suture. The syringe was inserted into a Kopf stereotactic electrode clamp mounting bracket attached to an electrode manipulator (David Kopf Instruments; Tujinga, CA) mounted on a Kopf stereotactic frame electrode A-P zeroing bar (#1450). Each mouse was positioned on the stereotactic frame and the needle inserted to the depth marker in the right cerebral hemisphere. Approximately 90–120 sec after injection of 5 μl, the needle was slowly withdrawn over the next 1 min. The burr hole was plugged with sterile bone wax and skin was closed with Tissumend surgical adhesive (Stryker Orthopedics; Kalamazoo, MI). The major endpoint in this study was animal survival; moribund animals that became unresponsive to mild external stimuli were euthanized and this date was used as an estimate of the date of death.

### Preparation of mitochondria and mitochondrial complex activities

Mitochondria were prepared according to Higuchi and Linn (42). Briefly, cells were washed twice in PBS. The pellet was resuspended in magnesium resuspension buffer (MgRSB; 10 mM NaCl, 1.5 mM MgCl_2_, 10 mM Tris-HCl, pH 7.5), incubated at 4°C for 10 min, and then disrupted with a Dounce glass homogenizer. The homogenate was diluted with 1.3 volumes of mannitol-sucrose buffer (MSB; 0.525 mM mannitol, 175 mM sucrose, 12.5 mM EDTA, 12.5 mM Tris-HCl, pH 7.5) and centrifuged at 1000 × g for 10 min to remove cell debris. The supernatant was further centrifuged at 20,000 × g for 20 min. The mitochondrial pellets were then digested with DNase I (Sigma-Aldrich) at 37°C for 30 min to digest nuclear DNA. After digestion, the mitochondrial pellets were washed three times with MSB, deep-frozen in liquid nitrogen, and stored at −80°C.

Mitochondrial complex activities and kinetic parameters for CcO activity were determined as previously described [50]. Purified CcO from bovine heart was acquired from Sigma-Aldrich (catalog # C5499, lot # 036M4111V). The purity and the identity of each band was confirmed by MS-based analysis [50, 77].

### Western blot analysis

Western blot analysis was performed as we previously described (11, 12). The following antibodies were used: anti-citrate synthase (1:1000 dilution, 16131-1-AP, ProteinTech Group, Chicago, IL); anti-COX4-1 (1:1000 dilution, ab14744, Abcam, Cambridge, MA), and anti-COX4-2 (1:1000 dilution, 11463-1-AP, ProteinTech Group). Anti-COX4-1 and anti-COX4-2 antibodies were tested for specificity, and no cross-reactivity between isoforms was detected.

### Cell cycle analysis

Cell cycle analysis was performed as we previously described (45). Briefly, glioma cells were cultured in 6-well plates and synchronized for 48 h in normal DMEM/F12 serum-free medium. After 48 h, serum-free medium was replaced by DMEM/F12 medium supplemented with 7% serum with or without graded concentrations of CPZ for 24 h. Then, cells were collected and pellets resuspended in 70% cold ethanol for up to 18 h. Fixed cells were pelleted by centrifugation and treated (20 min, room temperature) with 100 U of DNase-free RNase A/10^6^ cells. Nuclei were stained with 500 μl of propidium iodide (20 μg/ml) before nuclear DNA content was analyzed by flow cytometry using a BD Accuri™ C6 flow cytometer. All data were acquired and analyzed using FlowJo software.

### Molecular modeling

Structural model generation and molecular docking studies were conducted using the programs of the Schrödinger Suite 2015 (Schrödinger, LLC, New York, NY, 2015). Based on the crystal structures of mouse CcO (Protein Data Bank ID: 2Y69) [78], two human CcO homology models were constructed for COX4-1 and COX4-2 using the Prime program. Potential ligand binding sites were identified based on structural analysis of the CcO models using the SiteMap program. The three-dimensional structure of CPZ was prepared using the LigPrep program and the docking studies were conducted with the Glide program. Specifically, the induced-fit-docking (IFD) protocol [79], which is capable of sampling dramatic side-chain conformational changes as well as minor changes in protein backbone structure, was applied to explore the modes of CPZ binding at different binding sites. Residues within 5 Å of the docked CPZ were allowed to be flexible and the docked results were scored using the extra-precision (XP) mode of Glide.

### Statistics

Data were evaluated using GraphPad. All reported *p* values are two-sided *t*-test, and *p* values < 0.05 were considered to indicate statistical significance. Experiments were performed in triplicate and were performed twice or more to verify the results. Data are shown as the means ± S.D. *p* < 0.05 (*), *p* < 0.01 (**), *p* < 0.001 (***) and *p* < 0.0001 (****).
